# Organizational health climate as a precondition for health-oriented leadership: expanding the link between leadership and employee well-being

**DOI:** 10.3389/fpsyg.2023.1181599

**Published:** 2023-06-05

**Authors:** Friederike Teetzen, Katharina Klug, Holger Steinmetz, Sabine Gregersen

**Affiliations:** ^1^Department of Work and Organizational Psychology, Institute for Psychology, University of Hamburg, Hamburg, Germany; ^2^Faculty of Business Studies and Economics, University of Bremen, Bremen, Germany; ^3^Faculty of Management, University of Trier, Trier, Germany; ^4^Institution for Statutory Accident Insurance and Prevention in the Health and Welfare Services, Hamburg, Germany

**Keywords:** organizational health climate, health-oriented leadership, employee wellbeing, emotional exhaustion, job satisfaction, antecedents of leadership

## Abstract

The link between leadership and employee well-being is long established. In particular, health-oriented leadership is discussed as a leadership style specifically promoting employee well-being. However, the preconditions of health-oriented leadership remain largely unexplored. From the perspective of conservation of resources theory, leaders can only provide resources when receiving some themselves. We propose that organizational health climate (OHC) is an important organization-based resource for a health-oriented leadership style. More specifically, we hypothesize that the relationship between OHC and employee job satisfaction and emotional exhaustion is mediated by health-oriented leadership. We thereby differentiate two levels of analysis: a within-team level and a between-team level. We examined 74 teams with 423 employees of childcare centers at three time points, each 6 months apart. By means of multilevel structural equation modeling, we found OHC to be a significant antecedent of health-oriented leadership at the between-team level. The relationship between OHC and employee job satisfaction was mediated by health-oriented leadership at the between-team level, but not at the within-team level. The relationship between OHC and employee exhaustion showed another pattern of relationships at the different levels of analysis, while it was not significantly mediated by health-oriented leadership. This indicates the value of differentiating between levels of analysis. We discuss the implications for theory and practice that can be drawn from our findings.

## 1. Introduction

In the modern world, workplace well-being is an increasing concern for employees and organizations. Due to the intensification of work and increasing mental demands in many jobs, stress is becoming more prevalent, with potentially lasting consequences for employees’ health and quality of life (Sonnentag and Frese, 2002; de Jonge and Dorman, 2017). Additionally, work stress is among the most prevalent causes of sickness-related absence from work and is a highly cost-intensive issue for organizations. In 2020, the loss of gross value added due to sickness absence was estimated to be 144 billion euros in Germany (Brenscheidt et al., 2022), nearly 25% of which was due to psychological and stress diseases. These numbers are especially acute in the social care sector (e.g., education, child care and nursing), which have had, and continue to have, the highest sickness rates among all industrial sectors in Germany, resulting in significant costs and skill shortages (Kordt, 2014; Brenscheidt et al., 2022). Thus, the entire economy, and particularly the social care sector, require preventing stress and enhancing the well-being of employees to sustain employability in Germany, where our sample is situated.

Abundant research in the last 15 years has identified leadership as playing a significant role in employee well-being (Montano et al., 2017; Teetzen et al., 2022). Complementing this evidence, specific measures of health-oriented leadership have been formulated to acknowledge the leadership–well-being link, for example, the *health-oriented leadership* concept by Franke et al. (2014). It describes a comprehensive framework of attitudes and action patterns of leaders that enhance employee well-being and has great leverage in the improvement and maintenance of employee well-being (e.g., Vonderlin et al., 2021; Hauff et al., 2022). Moreover, such health-specific leadership concepts have been shown to contribute to employee well-being above and beyond what is considered generally constructive leadership (Gurt et al., 2011; Franke et al., 2014; Vincent-Höper and Stein, 2019; Kaluza et al., 2021). Therefore, to understand the mechanisms of how leaders influence employee health and well-being, it is important to consider their specific attitudes and behaviors toward health concerns at work through health-specific measurements.

However, the specific preconditions that leaders need to be able to lead in a health-oriented way and, thus, enhance employee well-being, have scarcely been researched (Alilyyani et al., 2018; Inceoglu et al., 2021), especially at the organizational level (for two exceptions, see Turgut et al., 2020; Krick et al., 2022). Previous research has largely focused on leaders’ or employees’ individual characteristics, behavior or job demands and resources (e.g., Arnold and Rigotti, 2020; Klug et al., 2022; Pischel et al., 2022). But leaders and employees are also embedded in organizational contexts that frame their behavioral scope (Oc, 2018). Neglecting organizational antecedents thus renders an incomplete picture of what is needed to promote healthy leadership. We suggest that the organizational climate, which defines the shared perceptions of organizational policies, practices, and procedures and their attached meaning to them (Loh et al., 2019), is a critical leadership precondition. More specifically, we believe that the *organizational health climate* (OHC, Zweber et al., 2016) provides a crucial antecedent for health-oriented leaders. OHC is a facet-specific climate measure that explicitly focuses on the psychological well-being of employees through the perceived organizations’ prioritization of employee health (Zohar and Luria, 2005; Zweber et al., 2015). It is largely driven by senior management (Dollard and Bakker, 2010) and provides cues about the kinds of behaviors that are expected and rewarded in healthy organizations (Dollard et al., 2019). This is comparable to the role of other climate facets as normative contexts, such as safety climate, which acts as a safety signal for directors and teachers in schools that they work in a safe environment and can behave in safety-enhancing ways (Yulita and Idris, 2017).

In our study, we draw on the conservation of resources theory (COR, Hobfoll, 1989) and argue that leaders in possession of resources are more likely to invest these in employees (Hobfoll et al., 2018). Based on that logic, several studies have found a positive influence on leader behavior by task-related or relational job resources, such as delegation, autonomy, and social support (e.g., Arnold and Rigotti, 2020; Krick et al., 2022); however, organizational-level resources have been widely neglected. This lack of consideration of organizational-level factors has been criticized by Hobfoll et al. (2018) and is needed to provide the optimal organizational environment for health-oriented leadership behaviors to enhance employee well-being. As one of the few examples, Krick et al. (2022) found organizational HRM strategies to be a valuable antecedent for health-oriented leadership. Consistent with that, we believe OHC acts as an organizational resource for leaders by which they acquire orientation and encouragement to act in a health-oriented way. Thus, we propose that OHC is a valuable organizational antecedent of health-oriented leadership.

Furthermore, while research has linked OHC to improved psychological health and reduced psychological strain (Zweber et al., 2015), the mechanisms behind this relationship remain unclear (e.g., Kaluza et al., 2020). Since leaders are the focal figures to transport organizational values and priorities to lower-level employees, we further propose that health-oriented leadership is a key mechanism by which a healthy organizational climate influences employee well-being. According to COR theory, different resources (like an OHC and health-oriented leadership) initiate a resource caravan passageway by reinforcing each other and, hence, replenish the resource reservoir of employees to enhance their well-being and leave them less susceptible to resource loss (Hobfoll, 2012).

While the outlined mediation process is important to be considered, it is not clear at which level (between teams vs. within teams) the proposed mechanisms mainly take place. The functioning of an organization as a whole depends on intergroup cooperation as well as on the functioning of teams (van Knippenberg, 2003). Thus, the shared perceptions inherent in OHC and health-oriented leadership at the between-team level may be grounded in different social processes than the individual within-team perceptions (Dollard et al., 2012a), which has valuable implications for leaders wanting to lead those teams. Thus, we differentiate the mediating mechanisms at the between-team and within-team levels.

**Figure 1:**
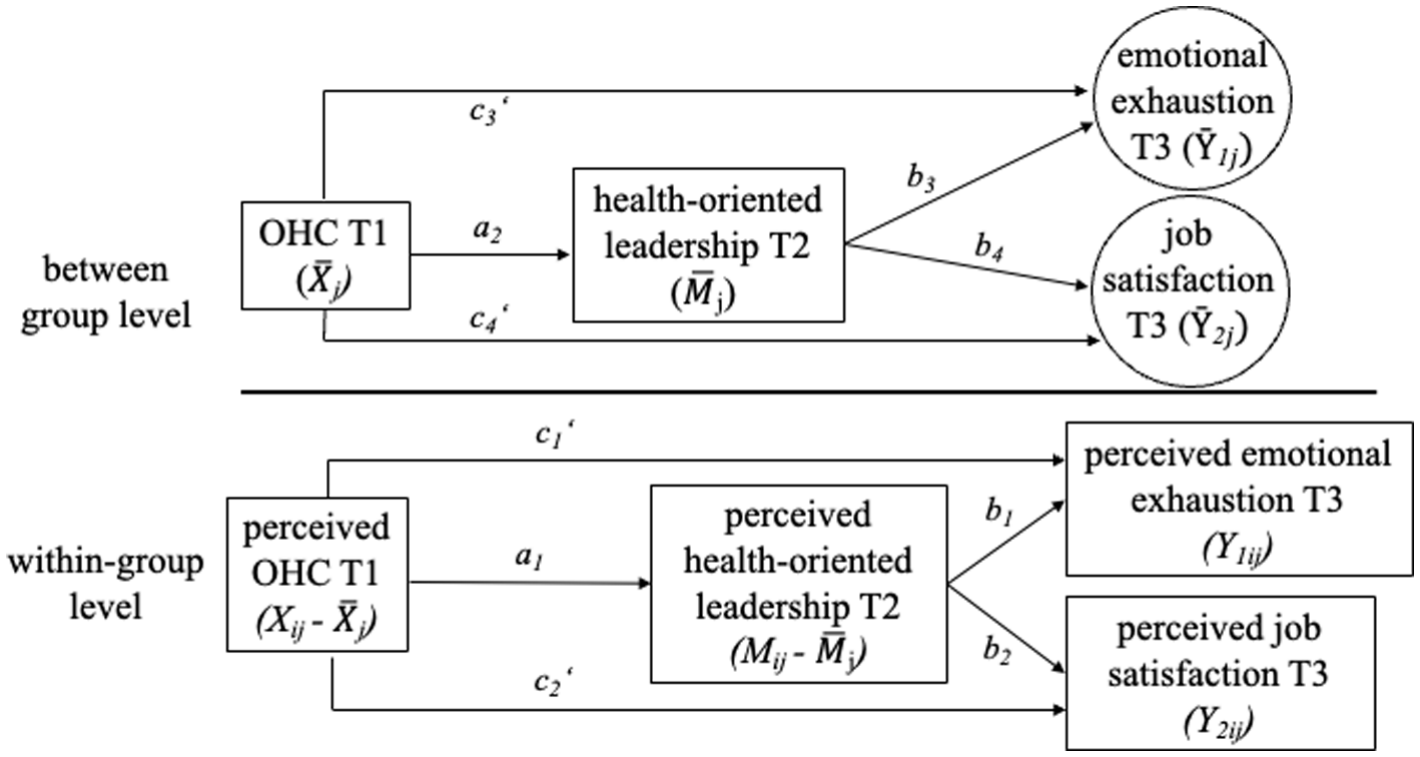
Proposed 2-2-1 mediation model ${\bar{X}}_{j}\;\mathrm{and}\;{\bar{M}}_{j}$ represent the aggregated OHC and health-oriented leadership of team *j*, respectively. *X_ij_*, *M_ij_*, *Y_1ij_*, *and Y_2ij_* represent the within-team OHC, health-oriented leadership, emotional exhaustion and job satisfaction of employee *i* in cluster *j*, respectively. For clarity, controls and autoregressive effect are not shown here.

Summing up, the present study has the following goals. First, by using a three-wave longitudinal survey, we examine OHC as an organizational antecedent of health-oriented leadership and analyze its role as a precondition for health-related leadership behavior. Second, we analyze the role of health-oriented leadership as a mediator of the OHC–employee well-being link. Third, we examine the mediating mechanism of health-oriented leadership at different levels of analysis (within and between teams) to reveal different relational patterns. We examine all these research goals in childcare centers, a unique context whose workforce well-being is in particular need of enhancing due to the tremendous impairments in this sector as outlined above. The proposed research model can be viewed in Figure 1.

Our study thereby contributes to existing research in several ways. First and foremost, we enhance knowledge of the supporting preconditions of health-oriented leadership, counteracting the mentioned omission of organizational antecedents of leadership with regard to employee well-being. By doing so, we attempt to broaden the scope to a more comprehensive framework that focuses on leaders and employees in the context in which they are embedded (Oc, 2018; Inceoglu et al., 2021). Contrary to personality (Tuncdogan et al., 2017) and leader ability (Courtright et al., 2016), organizational antecedents are more influenceable by organizations and can provide important starting points for supporting health-oriented behavior (Biron et al., 2018).

Second, we broaden the scope of the mediation processes of organizational climate. We thereby provide evidence for OHC as a distal factor to employee well-being and mediation via leadership, next to the more prominent mediators of job characteristics (e.g., Dollard et al., 2012a).

Finally, the differentiation of between-and within-team levels regarding the examined variables uncovers differences in relational patterns at different levels of analysis and advances theory and practice regarding the functioning of intergroup and intragroup dynamics. This can sensitize future research to differentiating levels of analysis.

## 2. Theory

### 2.1. Organizational health climate and health-oriented leadership

Research on organizational-level antecedents of leadership remains scarce (e.g., Sharma, 2018; Tafvelin et al., 2018). However, like employees, leaders also work in a contextual environment (Nielsen and Taris, 2019), which lays out the boundaries of the leadership playing field (Oc, 2018). Organizational climate establishes a framework for the desired and permitted behaviors of leaders in the workplace via their perceptions of implicit and explicit organizational policies and procedures (Hammer et al., 2019). However, a focus on the enhancement of employee well-being requires considering the organizational antecedents that improve this *particular* employee state. OHC is a facet-specific organizational climate that reflects organizational values and priorities regarding employee health and provides guidance for leadership behaviors via implicit norms and cues or explicit guidelines, thus functioning as an important leadership resource for enhancing employee well-being (Zohar, 2010; Zweber et al., 2015). Indeed, OHC is sensitive to improving employee well-being, as has been shown empirically by various studies (Zweber et al., 2015; Kaluza et al., 2020; Krick et al., 2022).

Health-oriented leadership is a specific facet of leadership that focuses on strengthening employee well-being and is clearly distinguishable from general leadership in influencing employee well-being (e.g., Gurt et al., 2011; Kaluza et al., 2021). It comprises two dimensions, StaffCare and SelfCare, which entail health-oriented values, awareness, and behaviors toward employees and leaders’ own health, respectively (Franke et al., 2014). In this study, we focus on the behavioral dimension of StaffCare and define health-oriented leadership as the activities and actions leaders take to improve employee well-being (i.e., designing the workplace for employees and supporting open communication).

To effectively enhance employee well-being, the health-specific organizational guidelines that an OHC provides must be enacted in a health-oriented fashion by focal members of the organization, i.e., leaders (Dimoff and Kelloway, 2017). This can only happen when the “espoused theory” of organizational climate, meaning the perceived OHC, will actually be translated into an “enacted theory,” meaning actual health-oriented leadership behaviors. This congruence between words and actions is grounded in the “theory of action,” which states that the values and beliefs of what we intend to do (espoused theory) guide what we actually do (enacted theory; Argyris and Schön, 1974). According to the “theory of planned behavior” (Ajzen, 1985), subjective norms are then responsible for showing this particular behavior. Subjective norms are a person’s belief that significant others approve of certain behaviors. Indeed, Biron et al. (2018) showed that organizational health climate was an antecedent of managerial quality in a cross-lagged panel of a sample of managers in four organizations. Similarly, Dollard et al. (2012a) found that OHC should be implemented as a starting condition in the work stress cascade, indicating “better readiness for change implementation” (Loh et al., 2021, p. S. 533). Moreover, Turgut et al. (2020) showed that leaders’ perceptions of subjective health norms in their organization were associated with their health-oriented leadership behaviour.

According to social exchange theory (Blau, 1964), leaders are more willing to show health-oriented leadership behaviors when the organization prioritizes employee well-being by providing organizational resources (Zohar and Luria, 2005; Zohar, 2010). Since corollary one of COR theory states that individuals in possession of resources are more likely to invest them, a resource in the form of OHC should make it more likely for health-oriented leaders to show health-oriented leadership behaviors and hand resources down to employees. This should be especially true for a facet-specific organizational resource such as OHC, since it sets the base for a greater congruence between words (OHC) and actions (health-oriented leadership) for employees (Zohar and Luria, 2005; Dollard et al., 2012b; Yulita and Idris, 2017; Biron et al., 2018). Dollard et al. (2012b) emphasize the importance of facet-specificity in terms of organizational climate, stating that “organizational climate constructs should be narrowly focused on the outcome of interest rather than broad bandwidth concepts” (p. 659). Indeed, the measurement of facet-specificity has been shown to increase the probability of detecting the desired behavior in various climate (e.g., Clarke, 2006). Thus, an OHC should promote leaders’ sense-making process in the direction of health orientation and should encourage health-oriented behaviors (Kaluza et al., 2020).

*H*1: OHC (at T1) is positively related to health-oriented leadership (at T2).

### 2.2. Health-oriented leadership and employee well-being

According to COR theory, stress occurs when employees’ resources (i.e., things or conditions they value) are lost or threatened. In turn, as people strive to protect and foster their resources, being able to maintain one’s resource level and gaining new resources has positive effects on well-being, according to the theory (Hobfoll, 1989; Hobfoll et al., 2018). Leaders influence the well-being of their employees through different pathways which reflect either the direct creation of resources or amplifying existing personal and organizational resources (Wegge et al., 2014).

For example, supporting behavior such as staff care can have direct effects on employees: By engaging in staff care, health-oriented leaders reduce stress and improve employees’ health by offering advice, support and showing concern for health at work. Accordingly, several studies support staff care as a resource for employees in the sense of COR theory, showing direct relationships with a range of health outcomes (Horstmann, 2018; Klug et al., 2019; Santa Maria et al., 2019; Arnold and Rigotti, 2020; Kaluza et al., 2021).

Leadership can also have indirect effects via fostering job-related or personal resources, both of which can reduce stress: Health-oriented leadership in terms of staff care includes leaders’ efforts to reduce stressors and create job resources for their employees by improving their teams’ work organization and work characteristics (Franke et al., 2014; Arnold, 2017). Empirical evidence supports job resources as an important mediating mechanism between leadership and employee health (Teetzen et al., 2022), and staff care has been shown to relate to work-related resources (Franke et al., 2014). Additionally, psychological capital, as a personal resource, has been shown to mediate the positive impact of staff care on employee health in the long run (Arnold and Rigotti, 2020).

Beyond dyadic interactions, leaders also act as role models for their employees (Wegge et al., 2014). By the extent to which leaders tend to their own and employees’ health, they set standards of acceptable and desirable behavior in their team. Via processes of social learning, employees may adopt similar behaviors for themselves (Dietz et al., 2020). A number of studies suggest that staff care facilitates employees’ own self care, thus setting off a process in which employees cultivate their own resources at work (Franke et al., 2014; Horstmann, 2018; Santa Maria et al., 2019; Klug et al., 2022).

In summary, both theoretical models and empirical evidence suggest that health-oriented leadership functions as a direct resource for employees, but also as a resource caravan in the sense that staff care creates the foundations to accumulate further job-related and personal resources (see Hobfoll et al., 2018). We hypothesize the following:

*H*2: Health-oriented leadership (at T2) is a) positively related to job satisfaction and b) negatively related to the emotional exhaustion of employees (at T3).

### 2.3. The mediation pathway between OHC and employee well-being via health-oriented leadership

Although research shows that OHC is positively related to employee well-being (e.g., Zweber et al., 2015), the mechanism between these two variables has seldom been studied (Schulz et al., 2017; Kaluza et al., 2020). The proposed mediation between OHC and employee well-being via health-oriented leadership is again based on COR theory (Hobfoll, 1989). The central idea of the theory is that people strive to obtain and preserve resources to acquire new ones. When multiple resources are gained, they travel in packs to form resource caravans (Hobfoll et al., 2018). For employees, the acquisition of multiple resources such as OHC and health-oriented leadership functions as a facet-specific resource caravan, which should promote job satisfaction. In case of emotional exhaustion, resource loss threats are high (Gorgievski and Hobfoll, 2008). In this situation, resource gain increases in importance to counteract resource loss (Hobfoll et al., 2018).

Dollard et al. (2019) stress that organizational climate affects worker psychological health by “shaping the social relations at work” (p. 10). This directly emphasizes the important role of leadership (Zohar and Luria, 2005), since leaders function as seminal figures and implementors of the organizational goals, priorities, and values and communicate which behaviors will be rewarded and which will be sanctioned (Dollard et al., 2019; Dietz et al., 2020). As Gurt et al. (2011) stated, an organization striving for health promotion must create a good fit between organizational values and leader behaviors to reach its desired goals.

Through the mechanisms outlined above, we expect the relationship between OHC and employee well-being to be mediated by health-oriented leadership:

*H*3: OHC (at T1) has a) positive indirect effects on job satisfaction (at T3) and b) negative indirect effects on emotional exhaustion (at T3) by the mediation of health-oriented leadership (at T2).

### 2.4. The different levels of analysis

Climate perceptions can be conceptualized at different levels: while *psychological climate* ascertains the sense-making process of an individual regarding his or her work environment (i.e., the individual level), the *group-level organizational climate* prescribes the “shared perceptions of employees on organizational policies, practices and procedures” (Loh et al., 2019, p. S. 443). While climate research becomes increasingly conducted at the group level because climate is often viewed as a group-level phenomenon, researchers on psychological climate have expressed the concern that individual differences in climate perceptions might be lost in this approach. This is why a *simultaneous* examination of these processes seems warranted (e.g., Schulz et al., 2017). Through that, organizational researchers gain knowledge of the empirical effect of “a comparison between individual and group levels of climate” (Loh et al., 2019, p. S. 444). Moreover, intergroup dynamics cannot be equalized to processes within groups, and this differentiation reveals different social processes that might take place (intergroup dynamics vs. within-team processes; van Knippenberg, 2003).

The differentiation of levels of analysis makes it possible to consider the grounding of variance in the criterion variables due to between-group effects (i.e., the team) and within-group effects (i.e., individual differences or social processes; Zhang et al., 2009) and provides evidence of which level of analysis is more relevant for the mediating mechanism of health-oriented leadership in the relationship between OHC and employee well-being. In the only study known to us that simultaneously examines within-team and between-team processes regarding OHC, Schulz et al. (2017) found between-team health climate to relate to several employee health outcomes beyond within-team health climate perceptions. Thus, in this study, we explicitly differentiate the mechanisms between and within teams regarding OHC and health-oriented leadership to draw implications for theory and practice as to different relational patterns between levels:

*Research Question 1*: Is the mediating pathway within teams of different strengths than that between teams?

## 3. Methods

### 3.1. Procedure

The data of this study were part of a larger research project on an intervention regarding supportive leadership in childcare centers in Germany. The objectives and usages of other studies of the dataset can be viewed in the supplemental material (Supplementary Table S1).

Invited to the data survey of the research project were leaders of 80 childcare centers and their teams. They had an overarching union, which oversaw the organizations’ health management system and steered the information policy regarding health-related topics via several division managers, newsletters and regular staff meetings. Data was gathered by means of a paper-pencil survey where all participants created their own code that allowed us to match individual responses to a team identifier. We collected data at three time points with time lags of 6 months between each data collection.

The leaders of part of the sample (30 leaders of 243 employees) participated in an intervention for supportive leadership training between T1 and T2. We conducted additional analyses to control for differences between the groups whose leaders had participated in the intervention and those whose leaders had not. These analyses gave no evidence of a difference between groups and can be viewed in the supplemental material (Supplementary Table S2).

### 3.2. Participants

Our final sample comprised 423 employees in 74 teams. From the originally invited 664 employees of 80 teams, 500 participants from 77 teams responded at T1, 362 participants from 74 teams responded at T2, and 321 participants from 70 teams responded at T3, yielding an average attrition rate of 41% over all time points. We excluded participants who (1) were trainees or interns to ensure a close enough working relationship with the leader and organization or (2) could not be matched to a team. We also excluded two teams due to insufficient team size (< 3 members). Team sizes ranged from 3 to 17 participants across all time points, with an average of 6.5. We compared participants who participated at T1 and T2 with those who participated only at T1 regarding OHC, health-oriented leadership, and employee well-being at T1 via t-tests. There were no differences between the subsamples found. We applied the same procedure to those participants who participated at T2 and T3 versus those only participating at T2 regarding the T2 variables. Again, no differences were found.

Due to the study focusing on child care centers, the final sample was 98% female and the participants were 17–65 years old, with 13% nonpedagogical personnel and 77% pedagogical personnel, 50% worked full time, 31% worked part-time with more than 20 h and 18% less than 20 h. The job tenure ranged between 1 and 44  years and employees worked between 1 and 6 years with their leaders.

### 3.3. Measures

We used questionnaires as the main data collection approach due to previous research pointing to an advantage of this type of data collection approach for the construct types we applied (Vonderlin et al., 2021) and the ease to collect large amounts of data and the feasibility for respondents to participate in the study (Martínez-Navalón et al., 2019).

The used items varied with regard to their type of dimensionality (e.g., agreement-styled dimensions, frequency-styled dimensions, and satisfaction-styled dimensions).

#### 3.3.1. Organizational health climate

We measured OHC perceptions of employees at T1 and T2 via six items that were adapted to the context of social care from Ducki (2000). To make the instrument fitting to the daily language of our context, we added expressions such as “*our union*” instead of “*our organization*” (response format: 5-point scale with 1 = *does not apply at all* to 5 = *applies very often*). A sample item was, “Our union attaches great importance to the well-being and health of its employees.”

#### 3.3.2. Health-oriented leadership

We measured health-oriented leadership via follower reports at T1 and T2 and used four behavior- and relationship-oriented items of the health-oriented leadership scale by Franke et al. (2014) with a 5-point answering scale [1 = (almost) never to 5 = (almost) always]. Sample items were, “My supervisor reduces stress through improvements in the area of work organization (e.g., setting priorities, ensuring undisturbed work, daily planning)” and “My supervisor ensures that everyone interacts positively.”

#### 3.3.3. Job satisfaction

Follower job satisfaction was assessed with six items from the Copenhagen Psychosocial Questionnaire (Kristensen, 2000) at T1 and T3 with a 5-point answering scale (1 = not at all satisfied to 5 = very satisfied). A sample item was, “In general, how pleased are you with your work?”

#### 3.3.4. Emotional exhaustion

We measured emotional exhaustion with five items from the Maslach Burnout Inventory (Maslach and Jackson, 1981) at T1 and T3. The responses were given on a 6-point scale (1 = never to 6 = often). A sample item was, “I feel burned out from my work.”

### 3.4. Data analyses

#### 3.4.1. Aggregation procedure

We followed the recommendations and practices in the multilevel literature (Mathieu and Luciano, 2019) and calculated several statistics to examine both within- and between-group variance to justify the aggregation of our data. While the literature discusses several ways of how team level measures can be connected to individual perceptions (Chan, 1998), our perspective was that of a direct consensus model, which requires a certain amount of individual agreement of the respective team-level construct. Regarding OHC, a one-way between-group ANOVA showed sufficient between-group variance (*F* (76, 414) = 1.59, *p* = 0.003) as well as within-group variability (Newman and Sin, 2020) with an average r_*wg*(*j*)_ = 0.73, reaching the recommended threshold of r_wg(*j*)_ > 0.70 (LeBreton and Senter, 2008). An ICC [1] of 0.08 at T1 showed a low but adequate variance between groups for the climate measure (Bliese, 2000). The ICC [2] was .37 at T1 and indicated low reliability; however, one should not refrain from conducting a multilevel analysis due to a low ICC 2 value (e.g., Aguinis et al., 2013). Since we observed both the Level-1 and Level-2 climate in this study and all other values were in the expected direction, we decided to proceed with the aggregation of the climate measure. For leadership, the between-group variance and within-group variability were adequate, indicated by a significant one-way between-group ANOVA (*F* (76, 408) = 3.36, *p* = 0.001) and an average r_*wg*(*j*)_ = 0.75. An ICC [1] = 0.30 and ICC [2] = 0.71 also indicated moderate variance between groups and good reliability of the group variable.

#### 3.4.2. Analyses of the results

Our data had a nested structure with employees nested in teams. The aggregated team variables (between-team OHC and between-team health-oriented leadership) were measured at Level 2, while job satisfaction and emotional exhaustion of employees and within-team predictors were measured at Level 1.

To reflect the nestedness, and thus, multilevel structure of our data and to test our hypotheses, we used multilevel structural equation modeling (MSEM, Preacher et al., 2011) with a maximum likelihood estimator. The MSEM approach separates within and between components of all variables and, thus, allows for distinct investigation of the direct and indirect effects at each level (Preacher et al., 2011). To test our hypotheses, we specified a 2-2-1 mediation model following the approach outlined by Hofmann and Gavin (1998). We group-mean centered the Level-1 variables and reintroduced the means of those variables back at Level 2. This approach allowed us to separately examine between-and within-group effects. To test indirect effects, we calculated Monte Carlo confidence intervals as recommended by Hayes (2017). Overall, we used the open source software R (R Core Team, 2022) for data management and preprocessing and Mplus (Muthén and Muthén, 1998) for fitting the multilevel models.

## 4. Results

Descriptive statistics, correlations, and reliabilities can be found in Table 1. All correlations were in the expected direction.

**Table 1:** Means, standard deviations, reliabilities, and correlations.

Variable	*M*	*SD*	1	2	3	4	5	6	7	8
1. OHC (T1)	2.94	0.95	*(0.95)*	0.50^**^	0.42^**^	0.39^**^	−0.51^**^	−0.39^**^	0.67^**^	0.44^**^
2. OHC (T2)	3.05	0.97	0.66^**^	*(0.95)*	0.21^**^	0.29^**^	−0.36^**^	−0.26^**^	0.33^**^	0.20^**^
3. HoL (T1)	3.55	0.96	0.38^**^	0.21^*^	*(0.86)*	0.72^**^	−0.44^**^	−0.09	0.62^**^	0.43^**^
4. HoL (T2)	3.57	0.89	0.37^**^	0.31^**^	0.66^**^	*(0.86)*	−0.33^**^	−0.16^**^	0.49^**^	0.42^**^
5. Exhaustion (T1)	3.56	1.31	−0.57^**^	−0.48^**^	−0.36^**^	−0.34^**^	*(0.94)*	0.50^**^	−0.69^**^	−0.53^**^
6. Exhaustion (T3)	3.69	1.30	−0.51^**^	−0.45^**^	−0.23^**^	−0.28^**^	0.79^**^	*(0.95)*	−0.41^**^	−0.66^**^
7. Job satisfaction (T1)	3.69	0.75	0.61^**^	0.48^**^	0.49^**^	0.41^**^	−0.69^**^	−0.59^**^	*(0.91)*	0.62^**^
8. Job satisfaction (T3)	3.67	0.73	0.53^**^	0.46^**^	0.48^**^	0.47^**^	−0.64^**^	−0.66^**^	0.78^**^	*(0.93)*

Within-level correlations (*N* = 423 employees) are below the diagonal and between-level correlations (74 teams) are above the diagonal; (*ω*) are given in parentheses along the diagonal. ^*^p < 0.05, ^**^p < 0.01.

### 4.1. Results of the multilevel structural equation model

**Figure 2:**
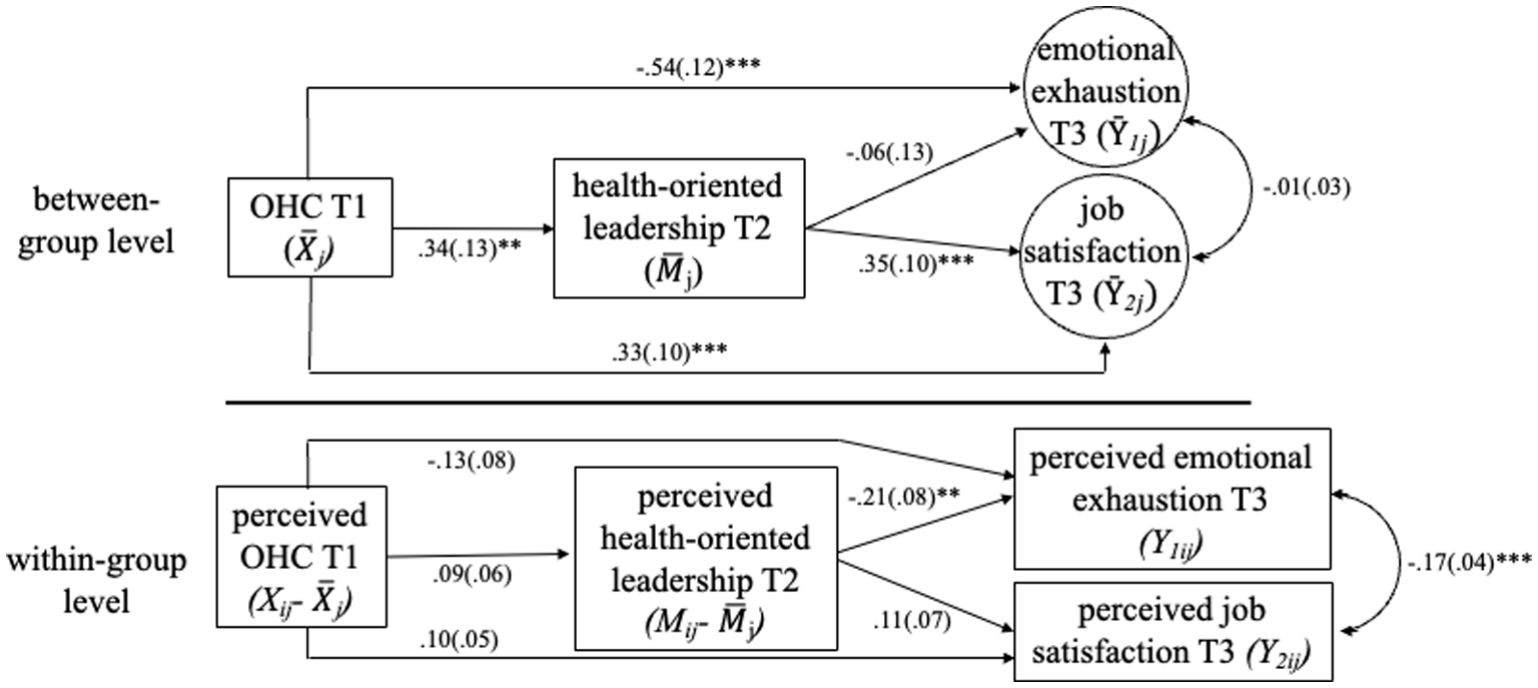
Results from the multilevel path model with unstandardized regression coefficients and standard errors in parentheses. Relationships with control variables and autoregressive effects can be viewed in Table 2. ^*^p < 0.05, ^**^p < 0.01, ^***^p < 0.001.

To test our hypotheses, we fitted the model indicated in Figure 2. The model showed a good fit to the data (*χ*^2^ = 8.478, *df* = 6, *p* = 0.21, *CFI* = 0.99, *TLI* = 0.97, *RMSEA* = 0.03). The effects of the model can be found in Table 2. As hypothesized, OHC at T1 had a positive effect on health-oriented leadership at T2 and was stronger between teams (*γ* = 0.34, *p* = 0.01, CI_95_ [0.08; 0.60]) than within teams (*γ* = 0.09, *p* = 0.12, CI_95_ [−0.02; 0.21]). This supports hypothesis H1 at the between-team level.

**Table 2:** Results of the multilevel structural equation models.

				Within/between group			𝛾(SE)		
Models	–2LL	∆df	p	𝜎^2^_HoL_	𝜎^2^_EE_	𝜎^2^_JS_	HoL (T2)	EE (T3)	JS (T3)
Unconditional model	1563.85		0.000	0.38/0.37	1.54/0.04	0.42/0.10			
*Within:*									
a_1_-path OHC (T1) → HoL (T2)	1542.76	1	0.000	0.35/0.37	1.54/0.04	0.42/0.10	0.09(0.06)		
c′_1_-path OHC (T1) → EE (T3)	1491.52	2	0.000	0.35/0.38	1.27/0.04			−0.13(0.08)	
c′_2_-path OHC (T1) → JS (T3)						0.33/0.11			0.10(0.05)
b_1_-path HoL (T2) → EE (T3)	1483.62	2	0.000	0.35/0.37	1.23/0.04			−0.21(0.08)^**^	
b_2_-path HoL(T2) → JS (T3)						0.31/0.11			0.11(0.07)
*Stabilities:*									
HoL (T1)	1293.82	3	0.000	0.30/0.38			0.34(0.06)^***^		
JS (T1)						0.20/0.11			60(0.05)^***^
EE (T1)					0.67/0.11			0.65(0.05)^***^	
*Between:*									
a_2_-path OHC (T1) → HoL (T2)	1288.23	1	0.000	0.30/0.35	0.67/0.11	0.20/0.11	0.34(0.13)^**^		
c′_3_-path OHC (T1) → EE (T3)	1261.87	2	0.000	0.30/0.35	0.66/0.04			−0.54(0.12)^***^	
c′_4_-path OHC (T1) → JS (T3)						0.19/0.06			33(0.10)^***^
b_3_-path HoL (T2) → EE (T3)	1242.09	2	0.000	0.30/0.35	0.66/0.03			−0.06(0.13)	
b_4_-path HoL(T2) → JS (T3)						0.20/0.03			0.35(0.10)^***^
*Within indirect effects*								𝛾 [MCCI_95_]
OHC (T1) → HoL (T2) → EE (T3; a_1_ × b_1_)							−0.02[−0.06; 0.003]		
OHC (T1) → HoL (T2) → JS (T3; a_1_ × b_2_)								0.01[−0.003; 0.04]	
*Between indirect effects*									
OHC (T1) → HoL (T2) → EE (T3; a_2_ × b_3_)							−0.02[−0.12;0.07]		
OHC (T1) → HoL (T2) → JS (T3; a_2_ × b_4_)									0.12[0.02;0.23]
*Difference test of within and between indirect effects of JS*									
Ind. JS within—ind. JS between								−0.11 [−0.22;-0.008]	

OHC = organizational health climate; HoL = health-oriented leadership; EE = employee emotional exhaustion; JS = employee job satisfaction; −2LL = −2^*^Log-Likelihood; ∆df = change in degrees of freedom; 𝜎^2^ = residual variance; and MCCI_95_ = Monte Carlo confidence intervals. Displayed are unstandardized estimates.

^*^p < 0.05, ^**^p < 0.01, ^***^p < 0.001.

Health-oriented leadership at T2 was related to job satisfaction at T3 between teams (*γ* = 0.35, *p* = 0.001, CI_95_ [0.16; 0.53]) but not within teams (*γ* = 0.11, *p* = 0.15, CI_95_ [−0.04; 0.25]) while being negatively related to emotional exhaustion within teams (*γ* = −0.21, *p* = 0.007, CI_95_ [−0.35; −0.06]), but not between teams (*γ* = −0.06, *p* = 0.63, CI_95_ [−0.31; 0.19]). Thus, hypothesis H2a was supported between 680 teams and H2b was supported within teams.

H3 asked for a mediation effect of OHC on (a) job satisfaction and (b) emotional exhaustion via health-oriented leadership and could only be supported for job satisfaction at the between-team level (*γ* = 0.12, *p* = 0.03, CI_95_ [0.01; 0.22]). The indirect within-team mediation effect was nonsignificant (*γ* = 0.01, *p* = 0.35, CI_95_ [−0.01; 0.03]). Thus, H3a was supported between teams. For emotional exhaustion, both the indirect between-team effect (*γ* = −0.02, *p* = 0.63, CI_95_ [−0.10; 0.06]) and the indirect within-team effect were nonsignificant (*γ* = −0.02, *p* = 0.23, CI_95_ [−0.05; 0.01]). Thus, H3b could not be supported.

Responding to Research Question 1, the mediation pathway between teams was significantly stronger than that within teams for the outcome of job satisfaction (*Diff*_*indJS*_ = *Ind*_*JSWithin*_ – *Ind*_*JSbetween*_ = 0.01–0.116 = −0.11, *p* = 0.04, *MCCI*_*95*_ [−0.22; −0.01].

## 5. Discussion

Our study aimed to identify organizational antecedents of health-oriented leadership and to explore the underlying mechanisms of the relationship between OHC and employee well-being in a facet-specific manner within and between teams in a longitudinal multilevel analysis with three measurement points. Our results showed that OHC can be viewed as an antecedent of health-oriented leadership at the between-team level. We also found health-oriented leadership to be an important mechanism by which OHC relates to job satisfaction of employees at the between-team level, with the effect being significantly stronger at the between-team than at the within-team level. We found no mediation effect of health-oriented leadership on the relationship between OHC and emotional exhaustion.

### 5.1. Theoretical implications

Recent research highlights the importance of examining relationships in their contextual environment (Hobfoll et al., 2018; Inceoglu et al., 2021). The larger organizational context must be considered when seeking to influence (health-oriented) behavior (e.g., Sharma, 2018). Thus, leaders need organizational prerequisites that support their way of leading for them to be effective and supportive. Corroborating this assumption and based on corollary one of the COR theory, hypothesis H1, linking OHC to health-oriented leadership, was supported at the between-team level. Thus, organizational climate functions as a resource for health-oriented leaders and grants them the opportunity to use and distribute those gained resources to their teams. Organizational climate thereby links the larger organizational context with the internal functioning of the organization (Dollard et al., 2019). With this finding, we contribute to the existing research by widening the lens to an important precondition of (health-oriented) leadership. This finding also contributes to the discussion of the ordering of the two variables of organizational climate and leadership. While initial research on organizational climate often conceptualized leaders as the creators of organizational climate (e.g., Clarke, 2013), recent research also identified organizational climate to be a plausible, if not necessary, precondition of leader actions (Yulita and Idris, 2017; Biron et al., 2018; Kaluza et al., 2020). Our analysis confirmed these studies and showed that the climate–leadership link is similarly plausible to the leadership–climate link. Future research must reveal if the ordering is reciprocal and if there are facet-specific differences for specific climate and leadership measures.

Our research further revealed that there is a difference in the OHC–health-oriented leadership relationship regarding different levels of analysis. A large share of the variance in health-oriented leadership is explained at the group level, which highlights the meaning of leadership for teams with regard to the conveyance of organizational climate.

In our second hypothesis, we postulated a positive influence of health-oriented leadership on employee well-being. While health-oriented leadership was positively related to job satisfaction at the between-team level, partially supporting H2a, its positive influence on emotional exhaustion (H2b) was only found at the within-team level, partially supporting H2b. The finding hints at the different mechanisms that influence employee well-being at the distinct levels of analysis (Wang and Howell, 2010): While job satisfaction is enhanced by positive influences of the whole team, for example, by improving team processes, appreciating the whole team for good work, or decreasing disruptive job demands for the team (Braun et al., 2013), emotional exhaustion seems to be a very individual perception that is instead based on the personal experience between the leader and the individual team members rather than on a group perception. It is not easy for leaders to consider all team members’ higher-level needs equally and, thus, influence their feelings of exhaustion in a similar fashion (Arnold, 2017). Corroborating this, studies have found lower ICCs for mental health than for other variables, suggesting that they are not significantly determined by group membership (Vonderlin et al., 2021). In sum, our differential findings on the different levels of analysis highlight the importance of examining level-specific mechanisms and outcomes.

Our mediation hypothesis regarding OHC and job satisfaction was supported at the between-team level, although not at the within-team level (partially supporting H3a). This finding identifies a group-level mechanism by which climate perceptions influence employee job satisfaction. Previous research showed that organizational factors influence employee behavior by a leader whose behavior is aligned with these organizational factors (Dietz et al., 2020). Thus, health-oriented leadership behaviors are a way through which health-related values and priorities of the organization trickle down to employees (Kaluza et al., 2020). This is in line with COR theory because employees “employ key resources not only to respond to stress but also to build a reservoir of sustaining resources for times of future need” (Hobfoll et al., 2018, p. 104). Thus, the distal organizational resource of OHC enhances the more proximal resource of health-oriented leadership, which creates a resource caravan passageway for employees and, thus, enhances job satisfaction (Hobfoll, 2012).

We further concretized our findings by showing that the mediating mechanism is stronger at the between-team level (answering Research Question 1), which corroborates research by Schulz et al. (2017). Leaders in childcare settings seem to emphasize the consequences and possibilities of OHC for their center, which transfers to employees focusing more on the “we” than the “I,” which results in greater job satisfaction, possibly by a higher identification with the work group (Riketta and van Dick, 2005). According to social identity theory (Ashforth and Mael, 1989), job satisfaction increases with the degree of identification with the organization because important human needs are met (van Dick and Haslam, 2012) and because a sharedness of values and norms by the group and shared group behaviors positively enhance individual outcomes (Häusser et al., 2020). This should be even more the case when organizational values and priorities and leadership behaviors are aligned in words and actions (Yulita and Idris, 2017). Individual perceptions in the team seem to fluctuate more easily, contingent upon the overall team atmosphere (Inceoglu et al., 2021).

The reason we did not find a significant mediation effect for emotional exhaustion (not supporting H3b) might have been the relatively high autoregressive effect of emotional exhaustion, indicating great inertia of emotional exhaustion within the measured timeframe (Hamaker and Grasman, 2015) and the already mentioned difficulty of attending to all employees equally in a group. Similar to our findings, Yulita and Idris (2017) found no significant relationship between enacted managerial support and emotional exhaustion. Moreover, previous research has shown that job demands, rather than job resources (such as health-oriented leadership) were the main predictors of emotional exhaustion (Dollard and Bakker, 2010; Dollard et al., 2012a).

### 5.2. Limitations and implications for future research

The results of our study must be seen in light of several limitations. Despite a multilevel design, which reduces common-method variance (Loh et al., 2019), we had a single-source design. Thus, we cannot rule out that common-method bias inflated the inspected relationships (Podsakoff et al., 2003). Even though one might intuitively point to a leader’s self-rated leadership measurement to acquaint a multisource design, previous research has shown that supervisors’ self-ratings of health-oriented leadership did not influence the relationship between employee ratings of health-oriented leadership and their mental distress, thus, consciously avoiding a leadership self-rating (Vonderlin et al., 2021). However, it would be valuable to integrate other-rated moderators to control for bias, for example leaders’ resources (i.e., skills; Pischel et al., 2022).

Furthermore, in terms of the ordering of the variables, our sample power did not suffice to integrate a cross-lagged panel model. Since previous research found evidence of the leadership–climate link (see Schneider et al., 2017) and the climate–leadership link (e.g., Biron et al., 2018), the direction of effects is not yet certain, and it could well be a reciprocal one. Thus, we need additional research to provide information on this topic. Future investigations should be especially sensitive to the facet-specificity of climate and leadership when aiming to explore the ordering of the variables (e.g., a leadership climate as a specific climate facet; Chen and Bliese, 2002). Further, OHC should also be examined as a moderator of (health-oriented) leadership as it has been identified in several works examining specific work conditions to identify the boundary conditions of health-oriented leadership (e.g., Dollard and Bakker, 2010; Law et al., 2011; Hall et al., 2013).

For the generalizability of the results, one has to keep in mind the industrial sector and the associated context (Inceoglu et al., 2021). Our study examined childcare centers and, thus, the social care context. However, for generalizability to other contexts, one must consider the potentially different mechanisms of conveying organizational climate and practicing health-oriented leadership in the organization (e.g., different communication patterns and collaboration schemes). Additionally, our sample was, due to the profession, our sample was very female-dominated. Thus, it would be valuable to examine moderators that indicate this gender distribution, for example, work–family conflict (e.g., Cinamon and Rich, 2002).

Furthermore, while we found differing results for the two levels of analysis, we did not test the nature of these differences. Future research should work on identifying the conditions on which these differences are grounded.

### 5.3. Practical implications

Interventions designed to improve employee mental health often focus on the individual or the personality and skills of the leader and not on organizational factors (Stuber et al., 2021). This circumstance involves the risk of ceiling effects when the skill set of a leader cannot be improved any further (Hammer et al., 2019). Our research provides support for the assumption that the value of employee job satisfaction is anchored in the organizational culture and that this can then be transferred to employees through leader behaviors (Parker et al., 2017; Health-oriented). Leadership behaviors can thereby be trained, as various previous research studies have shown (e.g., Parry and Sinha, 2005; Stein et al., 2021; Stuber et al., 2021). Thus, the organizational antecedents on which leader behavior forms must be considered when planning to influence leader behaviors (Nielsen and Miraglia, 2017).

Furthermore, knowledge about the social processes that influence how OHC and health-oriented leadership are perceived by employees is valuable to decide on the priorities in management behavior. Our research showed that employee job satisfaction is mainly influenced by a shared perception of the team regarding OHC and health-oriented leadership. Thus, addressing the team in a team-oriented way (e.g., providing information to the whole team, making decisions in participatory team meetings, providing transparency for the whole team) would be a good skill set to meet team needs regarding job satisfaction. At the same time, our study showed that impaired well-being (i.e., emotional exhaustion) cannot be influenced via health-oriented leadership at the team level. Thus, leaders can react more precisely to the needs of the team when they have a precise goal and familiar knowledge about the mechanisms by which OHC and health-oriented leadership are conveyed to reach this goal.

## 6. Conclusion

By identifying OHC as an organizational antecedent of health-oriented leadership, this study illustrates the relevance of organizational preconditions for the effective functioning of (health-oriented) leadership. This finding complements other research works that have identified the context as a notable part of leadership research (Oc, 2018; Sharma, 2018) and encourages research and practitioners to not solely focus on the outcomes of leadership but to also incorporate the “other side of the equation.” The study also showed that the relationship between OHC and job satisfaction is coming to life via health-oriented leadership and a team-based pathway, which gives organizations cues as to how to distribute OHC throughout the organizational environment. While research is starting to differentiate levels of analysis with greater frequency, the findings of this study encourage to a more consequent examination of multiple levels; otherwise, important knowledge on relationship patterns may be masked.

## Data availability statement

The raw data supporting the conclusions of this article will be made available by the authors upon request, with undue reservation.

## Ethics statement

The studies involving human participants were reviewed and approved by Institutional Review Board of the University of Hamburg. The patients/participants provided their written informed consent to participate in this study.

## Author contributions

FT, HS, and KK contributed to the conception and design of the study. FT organized the database, performed the statistical analysis, and wrote the first draft of the manuscript. All authors contributed to the article and approved the submitted version.

## Funding

We thank the Institution for statutory Accident Insurance and Prevention in the Health and Welfare Services for funding the open access publication fees. The funder was not involved in the study design and collection in the context of an evaluation, analysis or interpretation of the results.

## Conflict of interest

The authors declare that the research was conducted in the absence of any commercial or financial relationships that could be construed as a potential conflict of interest.

## Publisher’s note

All claims expressed in this article are solely those of the authors and do not necessarily represent those of their affiliated organizations, or those of the publisher, the editors and the reviewers. Any product that may be evaluated in this article, or claim that may be made by its manufacturer, is not guaranteed or endorsed by the publisher.
