# Supplementary material for: Organizational health climate as a precondition for health-oriented leadership: expanding the link between leadership and employee well-being
Source: Front Psychol. 2023 Jun 5;14:1181599. doi: 10.3389/fpsyg.2023.1181599 (PMC10277649; doi:10.3389/fpsyg.2023.1181599)
Supplement: Supplementary file 1 [file Table_1.DOCX]

Supplementary Material

**Organizational health climate as a precondition for health-oriented leadership: Expanding the link between leadership and employee well-being**

**Friederike Teetzen^1*^, Katharina Klug^2^†, Holger Steinmetz^3^†, Sabine Gregersen^4^, & Sylvie Vincent-Höper^1^**

^1^ Department of Work & Organizational Psychology, Institute for Psychology, University of Hamburg, Hamburg, Germany

^2^ Faculty of Business Studies and Economics, University of Bremen, Bremen, Germany

^3^ Faculty of Management, University of Trier, Germany

^4^ Institution for statutory Accident Insurance and Prevention in the Health and Welfare Services, Hamburg, Germany

† these authors contributed equally to this work

*** Correspondence:** Friederike Teetzen: friederike.teetzen@uni-hamburg.de

# Data transparency table of multiple articles published from the same dataset in the current study

| **Table S1.** Data transparency table of multiple articles published from the same dataset in the current study | | | |
| --- | --- | --- | --- |
|  | **This publication** | **Stein et al.**  **(2021, Journal of Occupational Health Psychology)** | **Stein et al.**  **(2020, Leadership & Organizational Development Journal)** |
| Study purpose | The examination of the organizational antecedents of health-oriented leadership and the mediating potential of health-oriented leadership in the relationship of organizational health climate and employee well-being with a differentiation within and between teams. | Cluster-randomized field trial examining the effect of supportive leadership training on employee social well-being and employee hedonic well-being. | This study positively links the workload of leaders to the emotional exhaustion of employees by constraining the enactment of social support by the leader. |
| Theories used | Conservation of Resources Theory, social identity theory. | Conservation of Resources Theory. | Theoretical work on social support, Conservation of Resources Theory. |
| Constructs/variables | Organizational health climate (T1), health-oriented leadership (T2), and job satisfaction and emotional exhaustion (T3). | Qualitative workload, quantitative workload (T1), LMX quality, emotional exhaustion, job satisfaction, and WHO-5 Well-Being Index (T1-T3). | Workload of leaders and employees, supportive leadership, and emotional exhaustion (T1). |
| Analysis techniques | Multilevel mediation analysis with structural equation modeling. | Cluster-randomized field trial, analyzed with linear mixed-effects models. | Multilevel regression analyses. |
| Results | Organizational health climate is an organizational antecedent of health-oriented leadership. There were no mediation effects found between organizational health climate and employee well-being via health-oriented leadership. However, the effect-patterns were very different for the different levels of analysis. | The relationship of LMX quality and emotional exhaustion varied depending on the baseline perceptions of employee quantitative workload. Those with high quantitative workload benefited most from the intervention. | Leader workload was negatively related to the employees’ perception of leader support, which in turn was positively related to employee exhaustion. |
| Theoretical implications | The organizational environment (such as organizational health climate) is an important and so far widely neglected precondition of health-oriented leadership. The study also strengthened the climate–leadership link. The differential results for the different levels of analysis give indication of variable social mechanisms that are at work at the respective levels and call for a more differentiated examination in multilevel work. | Examining a supportive leadership training from a conservation of resources theory perspective, this study showed that the focus on positive employee well-being units (i.e., LMX quality with the leader) should be promoted. It also showed that the training might not be equally effective for everyone but that there are conditions that influence the training effect (i.e., high quantitative workload). | Not only personal attributes, but also environmental factors (i.e., workload) influence the ability of leaders to show social support. Thus, the leaders’ work context represents a boundary condition to their ability to show supportive leadership behaviors. A multilevel lens of the conservation of resources theory provides knowledge on the senders of resources. |
| Practical implications | Organizations should foster organizational antecedents such as an organizational health climate to provide optimal conditions for and encourage health-oriented leadership. Congruence between organizational communication and leadership behavior seems to be important. | Supportive leadership trainings are an effective way to enhance employee well-being in organizations and LMX relationships between leaders and employees and are especially supportive for those employees with high quantitative workloads. | In addition to training leaders how to be supportive, organizations must widen their lenses to create circumstances under which leaders can also apply this knowledge. The work environment of leaders must be designed to meet these boundary conditions. |

# Supplementary analyses. Analyses of the difference between the group of employees whose leaders participated in a supportive leadership training and the group of employees whose leaders did not.

*Group sizes:*

Leaders who participated in the intervention (intervention group): 30 leaders of 243 employees; 🡪 of these, 16 leaders of 92 employees only participated in parts of the intervention.

Leaders, who did not participate in the intervention (control group): 47 leaders of 421 employees

(missing values: 10).

*Growth model of leadership with the predictor of “treatment condition” for the slope*:

We calculated growth curve models of the health-oriented leadership variable, with the condition of treatment as a predictor of this growth.

Model fit of the unconditional model: χ^2^ (1) = 0.14, p = .71, RMSEA =.00, SRMR = .01, CFI = 1.0, TLI = .1.01

Model fit of the conditional model: χ^2^ (4) = 7.35, p = .12, RMSEA =.07, SRMR = .07, CFI = .99, TLI = .98

| **S2 Table.** Growth model of leadership with the predictor “treatment condition” for the slope. | | |
| --- | --- | --- |
|  | Unconditional model  *estimate* | Conditional model  *estimate* |
| *Means* |  |  |
| Intercept | 3.57*** | 3.57*** |
| Slope | -.02 | -.02 |
|  |  |  |
| *Variances* |  |  |
| Intercept | .51*** | .61*** |
| Slope | -.02 | .03 |
|  |  |  |
| Treatment |  | .01 |
| *Note:* N = 172, treatment = intervention vs. control group, * < .05. ** < .01. *** < .001 | | |

Note: the calculations were based on N = 172 due to attrition in the leadership variable along the three time points.
